# Metabolic syndrome and its influence on clinical characteristics in bipolar disorder type 1: a cross-sectional study

**DOI:** 10.1192/j.eurpsy.2025.1077

**Published:** 2025-08-26

**Authors:** S. Ben Aissa, K. Razki, C. Najar, A. Larnaout, W. Melki

**Affiliations:** 1 Psychiatry, Razi hospital, Manouba, Tunisia

## Abstract

**Introduction:**

Metabolic syndrome (MS) is a prevalent condition in individuals with bipolar disorder (BD) and may influence the expression of psychiatric symptoms. Factors contributing to this association include limited access to physical healthcare, lifestyle influences related to psychiatric symptoms, and adverse effects of psychotropic medications.

**Objectives:**

To compare the clinical characteristics of patients with Bipolar Disorder Type 1 (BD-1) with and without metabolic syndrome (MS).

**Methods:**

This was a cross-sectional descriptive study conducted over a three-month period from August to October 2023 at the Psychiatric Service D of Razi Hospital in Tunisia. Patients diagnosed with Bipolar Disorder Type 1 according to the diagnostic criteria of the DSM-5, aged 18 years or older, and in a euthymic phase (confirmed by Hamilton Depression Rating Scale (HDRS) score <8 and Young Mania Rating Scale score <13) were included. Sociodemographic data, clinical features, and treatment information were initially collected from medical records and then verified and completed through direct interviews using a predefined information sheet and psychometric assessments. Metabolic syndrome was diagnosed according to the criteria of the International Diabetes Federation.

All participants provided informed consent before enrollment, and confidentiality of their personal and medical information was strictly maintained. The study protocol was approved by the Institutional Review Board (IRB) of Razi Hospital, ensuring that the research was conducted in accordance with ethical guidelines and principles of patient autonomy, beneficence, and non-maleficence. Participants were informed about the voluntary nature of their participation and their right to withdraw at any time without consequences. Data collection and storage adhered to strict privacy regulations to protect participants’ rights and ensure data security.

**Results:**

Forty patients were included in the study, of whom 28 had MS and 12 did not. The mean age of patients was 34.5 ± 8.2 years in the MS group and 32.1 ± 6.5 years in the non-MS group. Participants with MS had a significantly younger mean age of disorder onset (22.6 ± 8.25 years) compared to those without MS (27.9 ± 4.86 years) (p = 0.02). We found that the mean number of suicide attempts was significantly higher in the MS group (2.8) compared to the non-MS group (1.6) (p = 0.001). Anxiety comorbidity was significantly higher in patients with MS compared to those without MS (57% vs 35%, p < 10^-3).

**Conclusions:**

Our study underscore the importance of addressing metabolic syndrome in the management of patients with BD-1. Given the higher prevalence of metabolic syndrome in this population and its impact on clinical outcomes, interventions aimed at preventing and managing metabolic syndrome components such as obesity, diabetes, and dyslipidemia are crucial.

**Disclosure of Interest:**

None Declared

